# Non-alcoholic fatty liver disease (NAFLD) and the cardiovascular disease (CVD) risk categories in primary care: is there an association?

**DOI:** 10.1186/s12875-020-01306-7

**Published:** 2020-11-20

**Authors:** Hayatul Najaa Miptah, Anis Safura Ramli, Mariam Mohamad, Hilwati Hashim, Zahirah Tharek

**Affiliations:** 1 Department of Primary Care Medicine, Faculty of Medicine, Universiti Teknologi MARA (UiTM), Selayang Campus, Jalan Prima Selayang 7, 68100 Batu Caves, Selangor Malaysia; 2 Institute of Pathology, Laboratory and Forensic Medicine (I-PPerForM), Universiti Teknologi MARA (UiTM), Sungai Buloh Campus, Jalan Hospital, 47000 Sungai Buloh, Selangor Malaysia; 3 Department of Population Health and Preventive Medicine, Faculty of Medicine, Universiti Teknologi MARA (UiTM), Sungai Buloh Campus, Jalan Hospital, 47000 Sungai Buloh, Selangor Malaysia; 4 Department of Radiology, Faculty of Medicine, Universiti Teknologi MARA (UiTM), Sungai Buloh Campus, Jalan Hospital, 47000 Sungai Buloh, Selangor Malaysia

**Keywords:** Non-alcoholic fatty liver disease, Cardiovascular disease risk factor, Framingham risk score, Primary care

## Abstract

**Background:**

Non-alcoholic fatty liver disease (NAFLD) is an emerging novel cardiovascular disease (CVD) risk factor. It’s prevalence is increasing globally. However, there is paucity in the evidence showing the association between NAFLD and CVD risk in primary care setting. Therefore, the objectives of this study were to determine the prevalence and factors associated with NAFLD among patients with ≥1 risk factor for NAFLD or CVD attending primary care clinics.

**Methodology:**

A cross sectional study was conducted in two clinics at a university primary care centre. Patients aged ≥18 years with ≥1 risk factor for NAFLD or CVD were recruited. Participants with history of established liver disease or chronic alcohol use were excluded. Socio-demographics, clinical related data, anthropometric measurements and blood investigation results were recorded in a proforma. Diagnosis of NAFLD was made using abdominal ultrasound. The 10-year CVD risk was calculated using the general Framingham Risk Score (FRS). Multiple logistic regression (MLogR) was performed to identify independent factors associated with NAFLD.

**Results:**

A total of 263 participants were recruited. The mean age was 52.3 ± 14.7 years old. Male and female were equally distributed. Majority of the participants were Malays (79.8%). The overall prevalence of NAFLD was 54.4% (95%CI 48,60%). Participants in the high FRS category have higher prevalence of NAFLD (65.5%), followed by those in the moderate category (55.4%) and the low category (46.3%), *p* = 0.025. From MLogR, independent factors associated with NAFLD were being employed (OR = 2.44, 95%CI 1.26,4.70, *p* = 0.008), obesity with BMI ≥27.5 (OR = 2.89, 95%CI 1.21,6.91, *p* = 0.017), elevated fasting glucose ≥5.6 mmol/L (OR = 2.79, 95%CI 1.44,5.43, *p* = 0.002), ALT ≥34 U/L (OR = 3.70, 95%CI 1.85,7.44, *p* < 0.001) and high FRS category (OR = 2.82, 95%CI 1.28,6.23, *p* = 0.010).

**Conclusion:**

NAFLD is highly prevalent among patients with ≥1 risk factor for NAFLD or CVD in these primary care clinics. Patients who were obese, have elevated fasting glucose, elevated ALT and in the high FRS category were more likely to have NAFLD. This study underscores the importance of targeted screening for NAFLD in those with risk factors in primary care. Aggressive intervention must be executed in those with NAFLD in order to reduce CVD complications and risk of progression.

## Background

Non-alcoholic fatty liver disease (NAFLD) is defined by excessive fat accumulation in the form of triglycerides (steatosis) in the liver [1]. In the early stage, it is usually benign with no evidence of hepatocellular injury or fibrosis [2]. The definitive diagnosis is usually made by histology (liver biopsy) [3]. However, imaging such as ultrasound, computed tomography or magnetic resonance imaging (MRI) are non-invasive method which are widely used to detect NAFLD [4]. The importance of early detection of NAFLD is due to its potential to progress to liver inflammation, hepatocellular injury, fibrosis and end stage liver failure [5] in the presence of metabolic risk factors. Furthermore, it has been suggested that NAFLD is a novel cardiovascular disease (CVD) risk factor [6]. Several studies have shown that the 10-year probability of CVD events were higher in patients with NAFLD [7, 8]. It was observed that NAFLD patients had significantly higher prevalence of coronary, cerebrovascular and peripheral vascular disease compared to patients without NAFLD [9]. This has also been demonstrated in a meta-analysis which found that patients with NAFLD had a higher risk of fatal or non-fatal CVD events [10].

The overall global prevalence of NAFLD was reported to be 25.2% [3]. Whereas in Asia-Pacific region, the prevalence reported is comparable to the Western countries since NAFLD has also become a public health concern [1]. The prevalence in Asia ranges between 11.5% in Taiwan [11] to 30% in India [12] and 32.6% in Sri Lanka [13]. In Malaysia, previous study conducted among suburban population who came for health screening in a private hospital has reported a prevalence of 22.7% [14]. In other studies, the prevalence was reported as 50% among patients with diabetes [15] and 56.7% among patients with hypercholesterolaemia [16]. However, there is insufficient evidence in terms of prevalence of NAFLD in primary care settings in Malaysia as previous studies were conducted in private hospital and public hospital settings.

Current data from studies conducted from other countries regarding NAFLD and CVD risk assessment in primary care setting is still unclear and conflicting. There is also insufficient published data on the association between NAFLD and CVD risk in Malaysia.

The rising prevalence of NAFLD has been associated with the rising prevalence of potential risk factors mainly obesity and type 2 diabetes mellitus (T2DM) [1]. In a study reporting obese and severely obese patients undergoing bariatric surgery, > 95% of them have NAFLD [3]. While in patients with T2DM, the prevalence was as high as 60% [3]. Other factors associated with NAFLD include dyslipidaemia, hypertension and elevated liver enzymes namely alanine aminotransferase (ALT) and gamma-glutamyltransferase (GGT) [17, 18]. These clinical factors are in keeping with features of metabolic syndrome (MetS). Some literatures have suggested that NAFLD is the liver manifestation of MetS [18]. However, there is also insufficient data with regards to factors associated with NAFLD in the Malaysian primary care settings as previous studies were conducted in secondary care.

Therefore, the objectives of this study were to determine the prevalence and factors associated with NAFLD among patients with ≥1 risk factor for NAFLD or CVD attending primary care clinics.

## Methods

### Study design and population

**Fig. 1:**
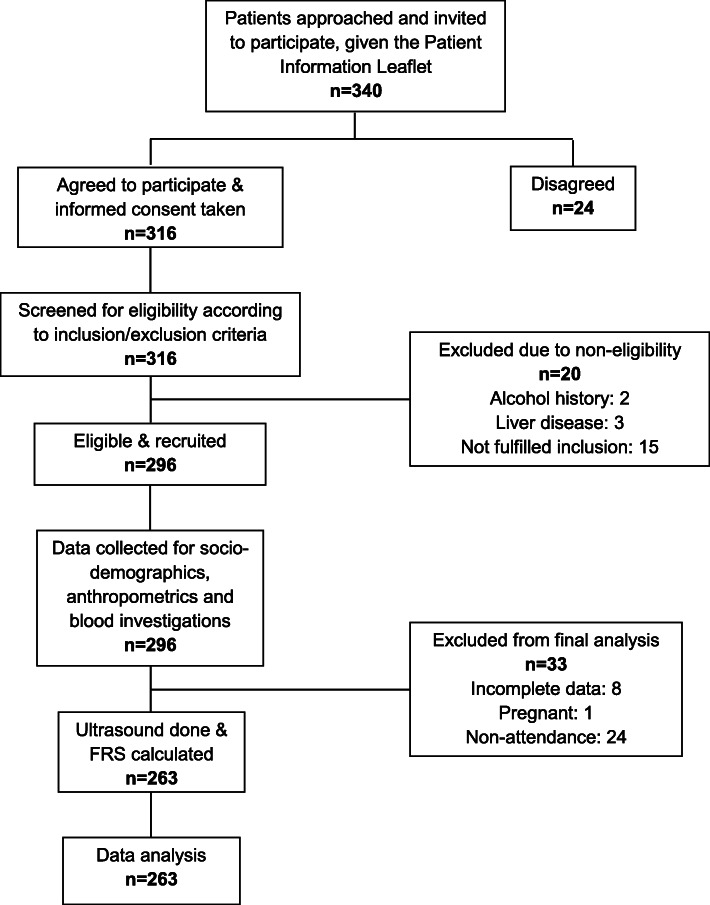
Flow chart of conduct of the study

We conducted a cross sectional study among patients attending two primary care clinics from a university health centre in Selangor, Malaysia between July 2017 to July 2018. The conduct of the study is shown in Fig. 1. We included participants 18 years and older who have at least one risk factors for NAFLD or CVD. These include abnormalities of liver enzymes (ALT ≥ 34 U/L, GGT > 60 U/L), cholesterol components (TC ≥ 5.0 mmol/L, LDL-C ≥ 3.1, TG ≥ 1.7 mmol/L, HDL-C ≤ 1.0 mmol/L in men or HDL-C ≤ 1.3 mmol/L in women), impaired fasting glucose > 5.6 mmol/L or random glucose > 7.8 mmol/L or known diabetes mellitus (DM), elevated HbA1c > 7.0%, elevated blood pressure (BP) ≥130/80 or known hypertension or abnormal waist circumference (WC) ≥80 cm in women or ≥ 90 cm in men. Patients with the following criteria were excluded: (a) previously diagnosed with chronic liver disease (b) history of alcohol intake of > 140 g per week for male and > 70 g per week for female and (c) pregnant prior to the study or during the study period.

### Study tool

#### Ultrasound

Diagnosis of NAFLD was made using ultrasound based on these criteria [19]: (a) liver echogenicity exceeded that of renal cortex and spleen; and there was attenuation of the ultrasound wave (b) visibility of the periportal echogenicity and intrahepatic architecture and (c) diaphragmatic echogenicity or loss of definition of the diaphragm. NAFLD was considered when any of the criteria above was present. If none was present, the diagnosis of NAFLD was excluded.

### Cardiovascular disease probability risk

The 10-year CVD risk for each participant was calculated using Framingham Risk Score (FRS) general CVD risk score 2008 [20]. It allows the calculation of probability risk of CVD events (coronary artery, cerebrovascular and peripheral vascular events). The score was calculated separately for men and women using online calculator. This tool has been calibrated and validated to be used in primary care settings among Malaysian adults [21]. The variables included in the tools were age, total cholesterol, HDL, systolic blood pressure, diabetes and smoking status. The score was transformed into 10-year CVD risk that categorized as low risk < 10%, moderate risk 10–20% and high risk > 20% [21].

### Sample size determination

Sample size was calculated using OpenEpi Tool Version 3.01, single proportion formula for “Sample Size for a Proportion or Descriptive Study” from http://openepi.com/SampleSize/SSPropor.htm

The formula for the calculation is:

$$ \boldsymbol{n}=\left[\mathbf{DEFF}\ast \mathbf{Np}\left(\mathbf{1}-\mathbf{p}\right)\right]/\left[\right({\boldsymbol{d}}^{\mathbf{2}}/{\boldsymbol{Z}}^{\mathbf{2}}\ \mathbf{1}-\boldsymbol{\upalpha} /\mathbf{2}\ast \left(\mathbf{N}-\mathbf{1}\right)+\mathbf{p}\ast \left(\mathbf{1}-\mathbf{p}\right)\Big] $$

n = sample size.

DEFF = design effect (for cluster surveys).

N = population size (for finite population correction factor or fpc).

p = hypothesized % frequency of outcome factor in the population.

*Z*^2^ 1 − α/2 = confidence interval.

d = desired precision.

The population size (N) was estimated based on the total number of patients receiving care in the primary care clinics in a year i.e. 8000 (as per registry from the information technology unit). The percentage (%) of the prevalence of NAFLD was estimated as 22.7% based on a previous study done in Malaysia [14]. In this study, the study population was adult patients attending health screening in a private hospital. In the absence of studies conducted in primary care setting, this is the closest to resemble primary care setting. Therefore, this percentage was taken as the hypothesized frequency. The confidence interval was taken as 95%. Based on these assumptions, the minimum required sample needed for this study was 261 patients. After considering additional 30% of non-eligibility and dropout rate, our study targeted to approach 339 patients.

### Sampling method and patient recruitment

Patients aged ≥18 years who attended the two primary care clinics during the data collection days were consecutively approached by the researcher in the waiting area after they have received their registration numbers. They were given a Patient Information Leaflet about the study in Malay or English languages and written informed consent was obtained. Patients who agreed were screened according to the eligibility criteria. These were done through physical examinations, tracing the medical records for medical history and blood investigations. Those who were eligible were recruited into the study.

### Data collection

Data was collected from July to September 2017 by the same researcher throughout the data collection period. One nurse from each clinic was trained on how to do the anthropometry measurement procedures according to the study protocol. This was to ensure standardisation and to minimise the variability of data collection. A structured proforma which consisted of six sections was used to collect data from participants. The six sections were (a) socio-demographic information (b) medical history (c) anthropometrics data (d) biochemistry results (e) ultrasound findings and (f) Framingham Risk Score.

### Study procedure

#### Anthropometric measurements

Weight and height were measured using Secca 767 digital scale while participants were wearing light clothing and without shoes. Weight was measured to a precision of 0.1 kg. Height was measured to a precision of 0.1 cm using the stadiometer from the same digital scale and converted to metres. BMI was calculated as per standard formula: BMI = weight (kg)/height (m^2^).

WC was measured to the nearest 0.1 cm using a non-stretchable measuring tape taken at the midpoint between the lower rib margin (12th rib) and the iliac crest. The measurement was repeated twice. If the difference of measurements were within 1 cm, the average was calculated. If the difference exceeded 1 cm, both measurements were repeated.

Blood pressure was measured using calibrated digital BP monitor, Omron-HEM-7111. Patients were ensured that they did not smoke, exercise, climb stairs or eat for at least 15 min prior to the BP measurements. BP was measured while patients seated upright with his/her right arm supported at the heart level. Measurements were taken twice at 2 min apart with the average of the first and second reading taken as the BP for analysis.

#### Blood investigations

Participants’ blood investigations which included fasting plasma glucose (FPG), fasting lipid profile (total cholesterol, triglyceride, LDL-cholesterol and HDL-cholesterol), liver enzymes (ALT and GGT) and HbA1c in patients with diabetes were collected. Data was obtained through the medical records if available within the last 3 months. If none was available, venous blood samples were taken from participants by trained nurses after an overnight fasting and sent to the same laboratory for analysis.

#### Ultrasound

Participants were given appointments to come for hepatic ultrasound, which was carried out by an experienced radiologist using a standard Phillips IU22 model with a curvilinear probe. Patients were asked to lie supine with the abdominal area exposed. Probe was placed at the right upper quadrant of abdomen to get the subcostal and intercostal views. The liver echogenicity, diaphragm and intrahepatic architecture were analysed.

#### Framingham risk score

All patients who completed ultrasound and the required blood investigations were stratified for CVD risk using the FRS online calculator.

### Data entry and statistical analysis

All the collected data were entered and analysed using Statistical Package for Social Sciences (SPSS) version 23 (SPSS, Inc., Chicago, IL, USA). Categorical variables were described in numbers and percentage whereas continuous variables were expressed as mean with standard deviation (SD). Inferential analysis was conducted to compare socio-demographic and clinical characteristics of participants with and without NAFLD. Simple logistic regression (SLogR) was used as preliminary analysis to identify the significant factors for NAFLD. Variables with *p* < 0.25 were included in the Multiple Logistic Regression (MLogR) to determine the independent associated factors for NAFLD after adjusting for the confounders. A *p*-value of < 0.05 was considered significant.

## Results

### Recruitment of participants

Out of 340 patients who were approached, 316 agreed to participate. Out of 316, 296 (87.1%) fulfilled the eligibility criteria. However, 33 were excluded from analysis due to not completing the ultrasound investigation giving reasons such as ‘busy with commitments’, ‘being overseas’, ‘no transportation’, or withdrew from the study; eight had incomplete blood investigation results and one participant was found to be pregnant during the reminder call for blood taking appointment. Therefore, the total number included in the final analysis was 263 (77.4%).

### Prevalence of NAFLD according to the socio-demographics and clinical characteristics

Out of 263 participants, 143 (54.4%) of them was found to have NAFLD with 95% CI (48,60%). Socio-demographics and clinical characteristics of those with and without NAFLD are shown in Table 1. Univariate analysis using Chi Square test found several factors to be significantly associated with NAFLD. These factors were age, gender, occupational status, T2DM, dyslipidaemia, BMI, waist circumference, blood pressure, fasting blood sugar, ALT, GGT and HDL-cholesterol.

**Table 1:** Prevalence of NAFLD according to the sociodemographic and clinical characteristics, (*N* = 263)

Variables	NAFLD Status*		Total, ***N*** = 263 (%)**	Chi-square^**a**^ (df)/t-test^**b**^ (df)	***P***-value***	OR (95% CI)/Mean diff (95% CI)^**b**^
Prevalence	Yes (*n* = 143, 54.4%)	No (*n* = 120, 45.6%)				
Age (years): (Mean ± SD)	53.31 (±13.34)	51.04 (±16.15)		-1.246^b^ (261)	0.214	−2.27 (− 5.85,1.31)^b^
Age Classification: n (%)						
18–29	7 (30.4)	16 (69.6)	23 (8.7)	11.97(4)^a^	**0.018**	1
30–39	21 (45.7)	25 (54.3)	46 (17.5)			0.37(0.14,0.96)
40–49	21 (72.4)	8 (27.6)	29 (11.0)			0.70(0.35,1.40)
50–59	32 (62.7)	19 (37.3)	51 (19.4)			2.20(0.90,5.38)
≥ 60	62 (54.4)	52 (45.6)	114 (43.4)			1.41(0.72,2.78)
Gender: n (%)						
Female	60 (45.8)	71 (54.2)	131 (49.8)			1
Male	83 (62.9)	49 (37.1)	132 (50.2)	7.729 (1)^a^	**0.005**	2.00(1.23,3.28)
Marital Status: n (%)						
Single	17 (58.5)	24 (41.5)	41 (15.6)	3.310 (2)^a^	0.192	1
Married	119 (56.9)	90 (43.1)	209 (79.5)			0.61(0.17,2.13)
Divorced/Widowed	7 (53.8)	6 (46.2)	13 (4.9)			1.13(0.37,3.49)
Ethnicity: n (%)						
Malay	111 (52.9)	99 (47.1)	210 (79.8)	2.865 (2)^a^	0.239	1
Chinese	22 (55)	18 (45.0)	40 (15.2)			1.09(0.55,2.15)
India/others	10 (76.9)	3 (23.1)	13 (5.0)			2.97(0.80,11,11)
Income group: n (%)						
B40 (≤ RM3000)	84 (54.2)	71(45.8)	155 (58.9)	0.005 (1)^a^	1.00	1
M40 + T20 (> RM3000)	59 (54.6)	49 (45.4)	108 (41.1)			1.018 (0.621,1.667)
Educational Level: n (%)						
No formal/primary	7 (41.2)	10 (58.8)	17 (6.5)	7.759 (3)^a^	0.051	1
Secondary	57 (60.6)	37 (39.4)	94 (35.7)			2.20(0.77,6.29)
Technical/Vocational/Diploma	35 (63.6)	20 (36.4)	55 (20.9)			2.50(0.82,7.59)
Tertiary	44 (45.4)	53 (54.6)	97 (36.9)			1.18(0.42,3.37)
Occupation: n (%)						
Unemployed	61 (48.0)	66 (52.0)	127 (48.3)	11.352 (3)^a^	**0.023**	1
Employed	82 (60.3)	54 (39.7)	136 (51.7)			0.61(0.37,0.99)
Smoking status n (%)						
Never	132 (53.7)	114 (46.3)	246 (93.5)	0.782 (1)^a^	0.455	1.58(0.57,4.42)
Active smoker	11 (64.7)	6 (35.3)	17 (6.5)			
Co-morbidities, n (%)						
T2DM						
Yes	63 (70.0)	27 (30.0)	90 (34.2)	13.468 (1)^a^	**< 0.001**	2.713(1.58,4.65)
No	80 (46.2)	93 (53.8)	173 (65.8)			
Dyslipidaemia						
Yes	92 (60.9)	59 (39.1)	151 (57.4)	6.14 (1)^a^	**0.017**	1.88(1.14,3.06)
No	51 (45.5)	61 (54.5)	112 (42.6)			
Hypertension						
Yes	79 (59.0)	55 (41.0)	134 (51.0)	2.31 (1)^a^	0.139	1.46(0.9,2.38)
No	64 (49.6)	65 (50.4)	129 (49.0)			
BMI, kg/m2: (Mean ± SD)	30.5 (±6.56)	27.52 (±5.87)		−3.947 (261)b	< 0.001	−3.06 (−4.58,-1.53)
BMI category: n (%)						
Not obese	11 (30.6)	25 (69.4)	36 (13.7)	23.316 (2)	**< 0.001**	1
Pre-obese	35 (41.2)	50 (58.8)	85 (32.3)			1.59(0.69,3.65)
Obese	97 (68.3)	45 (31.7)	142 (54.0)			4.90(2.22,10.82)
WC, cm: (Mean ± SD)	99.04 (±14.4)	90.71 (±11.57)		−5.099 (261)b	< 0,001	−8.329 (−11.547,-5.113)
WC category, n (%)						
Normal	19 (66.1)	37 (33.9)	56 (21.3)	11.99 (1)	**0.001**	2.909 (1.566,5.403)
Abnormal	124 (59.9)	83 (40.1)	207 (78.7)			
SBP: (Mean ± SD)	134.76 (±15.72)	127.99(±15.64)		−3.487 (261)^b^	0.001	−6.771 (−10.59,-2.94)^b^
SBP (mmHg) category: n (%)						
Normal < 130	49 (44.1)	62 (55.9)	111 (42.2)	8.1 (1)^a^	**0.006**	2.05 (1.25–3.37)
Elevated ≥130	94 (61.8)	58 (38.2)	152 (57.8)			
DBP: (Mean ± SD)	77.37 (±10.14)	74.05 (±10.08)		−2.652 (261)^b^	0.008	−3.32 (−5.79,-.086)^b^
DBP (mmHg) category: n (%)						
< 85, n (%)	110 (52.9)	98 (47.1)	208 (79.1)	0.89 (1)^a^	0.365	1.34 (0.73–2.45)
≥ 85, n (%)	33 (60.0)	22 (40.0)	55 (20.9)			
TC, mmol/L: (Mean ± SD)	4.83 (±1.18)	4.95 (±1.32)		0.764 (261)^b^	0.446	0.12 (−0.19,0.42)^b^
TC (mmol/L) category: n (%)						
< 5.0 mmol/L,	84 (54.9)	69 (45.1)	153 (58.2)	0.41 (1)^a^	0.900	0.95 (0.58–1.55)
≥ 5.0 mmol/L	59 (53.6)	51 (46.4)	110 (41.8)			
TG, mmol/L: (Mean ± SD)	1.62 (±0.82)	1.42 (±1.04)		−1.854 (261)b	0.065	−0.212 (− 0.437,0.013)^b^
TG (mmol/L) category: n (%)						
Normal < 1.7	91 (49.2)	94 (50.8)	185 (70.3)	6.76 (1)^a^	**0.01**	2.07 (1.19–3.59)
Elevated ≥1.7	52 (66.7)	26 (33.3)	78 (29.7)			
LDL-c, mmol/L: (Mean ± SD)	2.82 (±1.05)	2.89 (±1.14)		0.478 (261)^b^	0.633	0.064 (−0.201,0.330)^b^
LDL-c (mmol/L) category: n (%)						
Normal < 3.1	89 (54.3)	75 (45.7)	164 (62.4)	0.002 (1)^a^	1.0	1.01(0.61–1.67)
Elevated ≥3.1	54 (54.5)	45 (45.5)	99 (37.6)			
HDL-c, mmol/L: (Mean ± SD)	1.28 (±0.29)	1.41 (±0.34)		3.362 (261)^b^	**0.001**	0.131(0.054,0.208)^b^
HDL-c (mmol/L) category: n (%)						
Low ≤1.0 in men	12 (80.0)	3 (20.0)	15 (11.4)	1.62 (1)^a^	0.238	0.526 (0.194–1.430)
Normal > 1.0	71 (60.7)	46 (39.3)	117 (88.6)			
HDL-c (mmol/L) category: n (%)						
Low ≤1.3 in women	38 (67.9)	18 (32.1)	56 (21.3)	5.215 (1)^a^	0.024	0.488 (0.26–0.910)
Normal > 1.3	105 (50.7)	102 (49.3)	207 (78.7)			
FPG, mmol/L: (Mean ± SD)	6.833 (±4.30)	5.481 (±1.434)		−3.253 (256)^b^	**0.001**	−1.35 (−2.17,-0.53)^b^
FPG (mmol/L) category: n (%)						
Normal < 5.6	56 (40.6)	82 (59.4)	138 (53.5)	23.704 (1)^a^	**< 0.001**	3.55 (2.11–5.98)
Elevated ≥5.6	85 (70.8)	35 (29.2)	120 (46.5)			
HbA1c in %: (Mean ± SD)	7.63 (±1.62)	7.37 (±1.50)		−0.741 (98)^b^	0.461	−0.26 (−0.96,0.434)^b^
HbA1c category: n (%)						
Controlled < 7.0%	24 (75.0)	8 (25.0)	32 (32.0)	1.210 (1)^a^	0.812	0.80 (0.31–2.08)
Uncontrolled ≥7.0%	48 (70.6)	20 (29.4)	68 (68.0)			
ALT, U/L: (Mean ± SD)	35.69 (±28.61)	22.26 (±17.85)		−4.443 (258)^b^	< 0.001	−13.44 (−19.39, −7.48)^b^
ALT (U/L) category: n (%)						
Normal ≤34	84 (44.9)	103 (55.1)	187 (71.9)	23.26 (1)^a^	**< 0.001**	4.37 (2.34–8.16)
Elevated > 34	57 (78.1)	16 (21.9)	73 (28.1)			
GGT, U/L: (Mean ± SD)	48.95 (± 41.15)	36.16 (±44.6)		−2.398 (257)^b^	0.017	−12.79 (−23.29,-2.289)^b^
GGT (U/L) category: n (%)						
Normal ≤60	102 (49.3)	105 (50.7)	207 (79.9)	8.78 (1)^a^	**0.004**	2.69 (1.38–5.28)
Elevated > 60	38 (73.1)	14 (26.9)	52 (20.1)			

*SBP* Systolic blood pressure, *DBP* Diastolic blood pressure

*Data presented: as row percentage **Data presented as column percentage, *** significant at *p* < 0.05 ^a^Chi-square, ^b^Independent t-test

### Prevalence of NAFLD according to the FRS categories

**Fig. 2:**
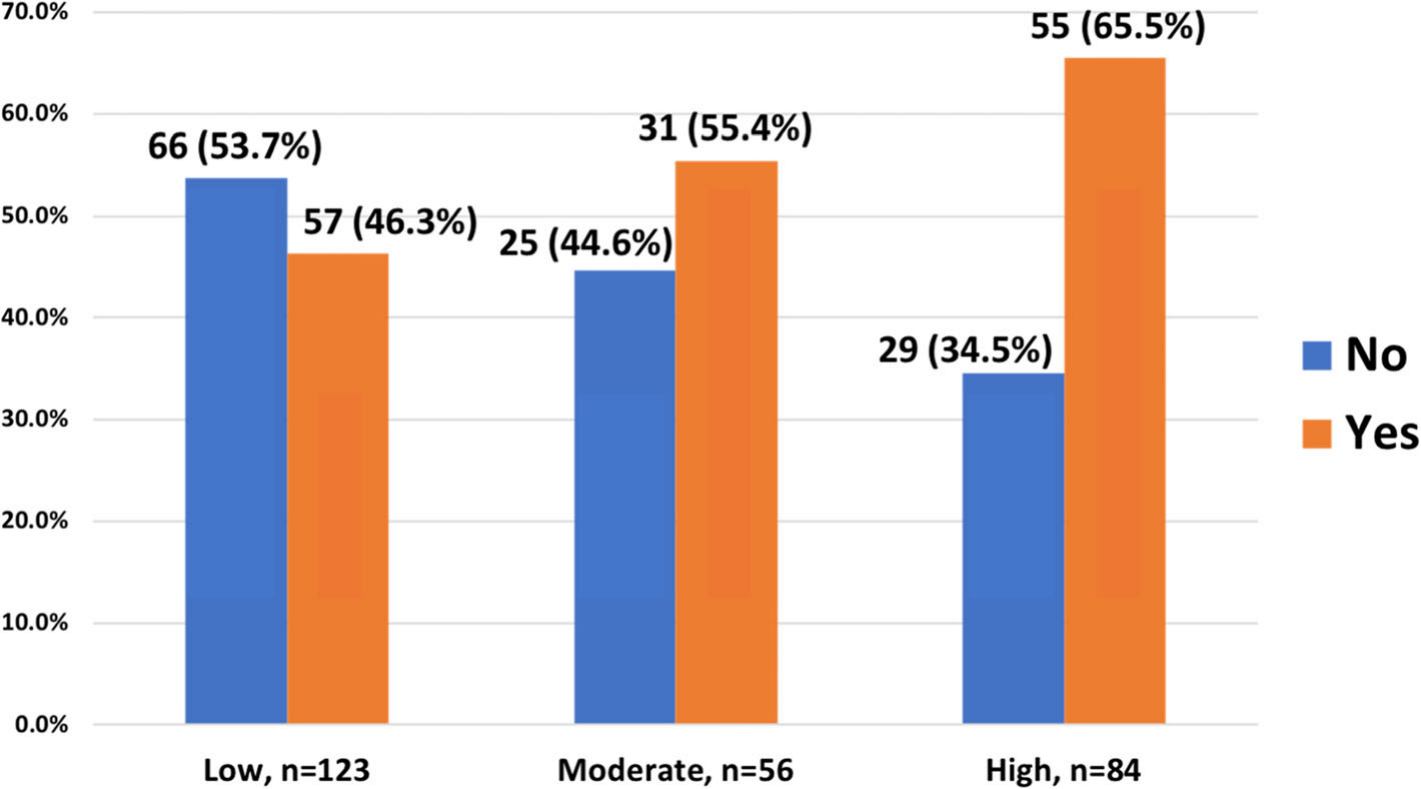
Prevalence of NAFLD according to the FRS category

Figure 2 illustrates the prevalence of NAFLD according to FRS categories. Participants with high FRS category had a greater prevalence of NAFLD (*p* = 0.025). The mean FRS was also calculated and compared between the two groups. It was found that the mean FRS was significantly higher in individuals with NAFLD compared to those without NAFLD (17.38 ± 12.35 vs. 12.35 ± 12.89, *p* = 0.003).

### Factors associated with NAFLD

Variables with *p*-value < 0.25 from SLogR were included in the MLogR. These included age, gender, employment status, BMI, WC, systolic blood pressure, HDL-C, FPG, ALT, GGT and FRS categories. Table 2 shows the results of the MLogR. Five factors were found to be independently associated with NAFLD. These were being employed (Adj. OR 2.44, 95%CI 1.46,4.70), BMI of obese category (Adj. OR 2.89, 95%CI 1.21,6.91), elevated FPG (Adj OR 2.79, 95%CI 1.44,5.43), elevated ALT (Adj OR 3.70, 95%CI 1.85,7.44) and high FRS category (Adj OR 2.82, 95%CI 1.28,6.23). Post regression analysis showed that the model reasonably fits well. The receiver operating characteristic (ROC) curve gave an area under the curve of 0.779 (95%CI 0.723,0.835) which indicated that the model could accurately discriminate 77.9% of the cases.

**Table 2:** Factors independently associated with NAFLD (MLogR)

Variables	Adj Beta (SE)	Wald (df)	Adj. OR (95%CI)	***P***-value
Occupational sector:		–	–	–
Not working	REF		1.00	
Working	0.89 (0.335)	7.071 (1)	2.44 (1.26,4.70)	**0.008**
BMI:				
Not-obese	REF		1.00	
Obese	1.060 (0.445)	5.679 (1)	2.89 (1.21,6.91)	**0.017**
FPG				
< 5.6 mmol/L	REF		1.00	
≥ 5.6 mmol/L	1.027 (0.339)	9.169 (1)	2.79 (1.44,5.43)	**0.002**
ALT				
≤ 34 U/L	REF		1.00	
> 34 U/L	1.310 (0.355)	13.587 (1)	3.70 (1.85, 7.44)	**< 0.001**
FRS category				
Low	REF		1.00	
Moderate	0.388 (0.413)	0.884 (1)	1.47 (0.66,3.31)	0.347
High	1.038 (0.403)	6.620 (1)	2.82 (1.28,6.23)	**0.010**

Notes:

*OR* Odds Ratio, *CI* Confidence interval, *df* Degree of freedom, *REF* Reference group

The model reasonably fits well (Hosmer-Lemeshow test: *p* = 0.168)

Model assumptions were met

No significant interactions and multicollinearity problem

Model explained between 23.1% (Cox and Snell R Square) and 30.8% (Nagelkerke R Square) of the variance in NAFLD group and correctly classified 73.4% of cases

## Discussion

### Main findings of the study and comparison with previous literature

To the best of our knowledge, this study was the first prospective study in Malaysia to determine the prevalence of NAFLD in the primary care setting. Previous studies were conducted in secondary care settings. This study was also the first that has established the association of 10-year CVD risk categories and NAFLD in Malaysia.

In this study, the overall prevalence of NAFLD was reported as 54.4% (95% CI 48, 60%). Our finding is comparable with other local studies conducted among individuals with risk factors. The prevalence of NAFLD among participants with T2DM in a university hospital setting was 49.6% [15]. Our finding is also comparable to another study among participants with dyslipidaemia in another university hospital setting where the prevalence was 56.7% [16]. The prevalence of NAFLD among patients with MetS was even higher (82.8%) as reported by another study in a public hospital [22]. However, the prevalence of NAFLD in a general population attending health screening at a private hospital was lower at 22.7% compared to our study [14]. This is understandable as their study included individuals with and without risk factors for NAFLD or CVD.

In comparison with studies in the Asian region and the West, the prevalence of NAFLD in our study was also comparable with studies among individuals with risk factors. In India, the prevalence was 56.5% among individuals with T2DM attending diabetic clinics [23]. This was supported by a meta-analysis of 17 studies on the prevalence of NAFLD in patients with T2DM where the overall prevalence was 54% (95%CI 45,64%) [24]. In another study conducted among patients with T2DM in a Brazilian tertiary hospital, the prevalence was 69.4% [25]. In contrast to our study, the prevalence of NAFLD in the general population was found to be generally lower. A population-based cross sectional study in Thailand showed a prevalence of 22.9% [26]. In the US, the prevalence was 31% in a multi-ethnic population study [27]. Another population study in Italy showed that the prevalence of NAFLD was 22.6% [28]. Overall, the prevalence of NAFLD in our study which included individuals with at least 1 risk factor for NAFLD or CVD was shown to be higher when compared to studies done in the general population. Our finding was comparable to studies which included individuals with risk factors.

With regards to FRS categories, this study showed that the prevalence of NAFLD in the high FRS risk group was significantly higher compared to the moderate and low risk groups (65.5% vs. 55.4% vs. 46.3%, *p* = 0.025). Out of 143 participants with NAFLD, 39.9, 21.6 and 38.5% were in the low, moderate and high risk categories, respectively. Our findings were comparable to a study in Korea which showed that out of 5769 participants with NAFLD; 62.1, 28.4 and 9.5% were in the low, moderate and high risk categories, respectively [29]. However, their study used the FRS 10-year coronary artery disease (CAD) risk prediction tool.

Regarding factors associated with NAFLD, our study found five factors to be significantly associated. These included being employed, BMI of obese category, elevated FPG, elevated ALT and high FRS category.

Participants who were currently employed have a higher odd of having NAFLD (Adj. OR 2.44, 95% CI 1.46, 4.70). In contrast, a study in China reported that those who were employed were less likely to have NAFLD (OR 0.69, *p* = 0.008) [30]. Another study conducted among police officers in China found that high occupational stress (HR = 1.727, 95% CI = 1.405–2.124) and high personal strain (HR = 3.602, 95% CI = 1.912–6.787)) were independent predictors for NAFLD [31]. However, in our study, data was not collected with regards to the type of occupation. Therefore, further deduction could not be made and more studies are needed to explore the type of occupation which may be associated with NAFLD.

With regards to BMI, our study shows that participants who were obese (BMI ≥ 27.5 kg/m^2^) had 2.89 times the odds of having NAFLD compared to non-obese individuals (Adj. OR 2.89, 95%CI 1.21, 6.91). Similar finding was also observed from an Asian study which reported that overweight individuals (BMI > 25 kg/m^2^) were more likely to have NAFLD; OR 1.05 (95%CI 1.004,1.09) *p* = 0.031 [32]. In a study conducted in Malaysia among overweight individuals with BMI > 23 kg/m^2^, the odds of having NAFLD was also higher compared to those with normal BMI; OR 14.66 (95%CI 9.62,22.33) *p* < 0.001 [14]. These findings suggested that obese and overweight individuals are more likely to have NAFLD. This might be explained by the increased in fatty acid metabolism in overweight and obesity that lead to accumulation of triglyceride in the liver [33].

In our study, individuals with elevated FPG (≥ 5.6 mmol/L) had 2.79 times the odds of having NAFLD compared to those with normal FPG (Adj OR 2.79, 95%CI 1.44, 5.43). Similarly, in Sri Lanka, elevated FPG of ≥ 5.6 mmol/L was found to be associated with NAFLD; OR 1.7 (95%CI 1.39,2.08) *p* < 0.001 [13]. This is also comparable to a study in China which showed that elevated FPG (≥6.1 mmol/L) was independently associated with NAFLD; OR 3.324 (95%CI 1.89,5.85; *p* < 0.001) [34]. Elevated FPG indicates abnormal glucose metabolism or insulin resistance. This condition has been thought to be the influencing factor or active metabolic factor for fat deposition in the liver [2].

It was found from our study that those individuals with elevated ALT (> 34 U/L) had an increased odds of having NAFLD by 3.70 times compared to those with normal ALT level (Adj OR 3.70, 95%CI 1.85, 7.44). This is consistent with several studies which found that elevated ALT was significantly associated with NAFLD with slightly different upper limit values as per local references. In Taiwan, ALT (> 40 IU/L) was significantly associated with NAFLD; OR 5.66 (95%CI 3.99,8.01) *p* < 0.001 [11]. While in Sri Lanka, individuals with high ALT (defined as twice the upper limit of normal) had increased odds of having NAFLD compared to those with normal ALT level; OR 2.28 (95%CI 1.32,3.94) *p* = 0.003 [13].

In terms of CVD risk categories, our study shows that individuals in the high FRS CVD risk category had an increased odd of having NAFLD by 2.82 times compared to those in the low risk category (Adj OR 2.82, 95%CI 1.28,6.23). The study by Choi et al. found that NAFLD was strongly associated with moderate FRS CAD risk category (OR: 1.26 95%CI 1.11,1.42, p < 0.001) and high FRS CAD risk category (OR: 1.35 95%CI 1.10,1.65, p < 0.001) [29]. In a longitudinal cohort study in the US, multivariate analysis showed that FRS was the only variable significantly associated with new onset CAD (OR = 1.13, 95% CI = 1.05–1.21; *p* = 0.001) [7]. Patients with NAFLD was found to have a higher 10-year CAD risk than the general population of the same age and gender [7] . Therefore, the findings of this study underscore the significant association between NAFLD and CVD risk.

### Strengths and limitations

The strength of this study includes prospective diagnosis of NAFLD using ultrasound scan by a radiologist. This study was also the first that has established the association of 10-year CVD risk categories and NAFLD in Malaysia.

There are a few limitations of this study. Firstly, the cross-sectional study design would not be able to draw causal relationship between various factors associated with NAFLD. Secondly, the use of non-probability sampling method may be prone to sampling bias. Therefore, efforts were made to reduce sampling bias by ensuring patients who attended the clinics on data collection days were approached consecutively and invited to participate. Thirdly, this study was conducted in a university primary care setting where majority of the participants were Malays. Therefore, the results may not be generalised to other primary care settings where the population served might be different. It is also acknowledged that medications status was not highlighted in this study hence it might contribute to possible confounding factor to the final analysis. Finally, this study only included two main categories of factors that are associated with NAFLD i.e. the socio-demographics and clinical factors. Other factors that may be associated with NAFLD such as psychological and environmental factors were not included in this study. Therefore, the results of the multivariate analysis should be interpreted with caution.

### Potential clinical implications

This study purposely recruited patients with ≥ 1 risk factors for NAFLD or CVD rather than those with no risk factor. These patients were chosen because they should be targeted for screening of NAFLD in view of limited resources in the primary care clinics. Currently, there is no guideline on routine screening for NAFLD even in targeted groups. According to the American Association of the Study for Liver Disease (AASLD), recommendation for routine screening for NAFLD is not made at this point as there are still ongoing studies to better understand NAFLD in terms of its natural history, diagnosis and treatment [3]. However, the findings of our study should strengthen the body of evidence to suggest targeted screening for NAFLD in individuals with obesity, elevated FPG, elevated ALT and high FRS category.

**Fig. 3:**
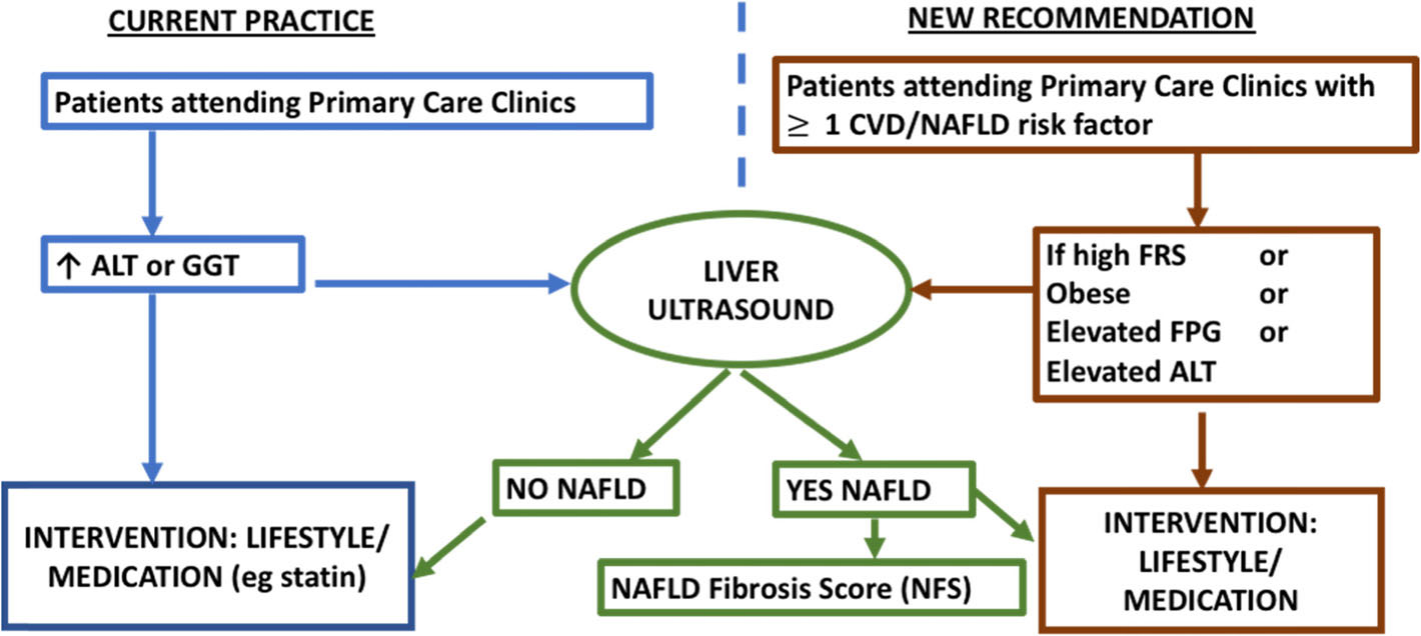
Proposed algorithm for screening of NAFLD in the target groups in Primary Care

Figure 3 illustrates the proposed algorithm for screening of NAFLD in the target groups.

Patients with at least one CVD or NAFLD risk factor should be risk stratified using the 10-year FRS general CVD risk score. If they are found to have high FRS, or obese or have elevated FPG or elevated ALT, they are recommended to have a liver ultrasound to screen for NAFLD. If the patients are found to have NAFLD, then the severity of the condition should be assessed using scoring such as NAFLD Fibrosis Score (NFS) to identify those who might be referred for liver biopsy. Regardless of their NAFLD status or NFS score, these patients should be targeted for aggressive lifestyle intervention and risk factor management.

## Conclusion

As a conclusion, this was the first study in Malaysia to document on the prevalence of NAFLD and associated factors in primary care setting. It has been demonstrated from this study that NAFLD is highly prevalent in patients with at least one risk factor for NAFLD/CVD in our primary care setting. This study has also established the significant association between NAFLD and high FRS risk category. Therefore, it is a pivotal to include patients in high FRS category as well as those who are obese, have elevated FPG and ALT in the consideration of NAFLD screening. Aggressive interventions must be targeted in those with NAFLD in order to reduce CVD complications and risk of progression to a more advanced liver disease. These interventions include lifestyle modification and risk factor management.

## Data Availability

Data are kept at the Institute of Pathology, Laboratory and Forensic Medicine (I-PPerForM), Universiti Teknologi MARA (UiTM), Sungai Buloh Campus, Jalan Hospital, 47000 Sungai Buloh, Selangor, Malaysia. Data will be shared by the corresponding author upon request and it is subjected to the data protection regulations.

## Abbreviations

AASLD: American Association of the Study for Liver Disease
Adj OR: Adjusted odds ratio
ALT: Alanine aminotransferase
BMI: Body mass index
BP: Blood pressure
CAD: Coronary artery disease
CVD: Cardiovascular disease
FPG: Fasting plasma glucose
FRS: Framingham Risk Score
GGT: Gamma-glutamyltransferase
MeTS: Metabolic syndrome
MLogR: Multiple logistic regression
MRI: Magnetic resonance imaging
NAFLD: Non-alcoholic fatty liver disease
NFS: NAFLD fibrosis score
RM: Ringgit Malaysia
ROC: Receiver operating characteristic
SD: Standard deviation
SLogR: Simple logistic regression
SPSS: Statistical Package for Social Sciences
T2DM: Type 2 diabetes mellitus
WC: Waist circumference
